# Development and Validation of an In Vitro Ocular Irritation Test for Ophthalmic Medical Devices with a Novel Reconstructed Human Corneal Epithelium Model

**DOI:** 10.3390/bioengineering13050545

**Published:** 2026-05-11

**Authors:** Payal Rawat, Umberto Rodella, Stefania D’Agostino, Eugenio Ragazzi, Orietta Rossi, Claudio Gatto, Laura Giurgola, Jana D’Amato Tóthová

**Affiliations:** 1 Research and Development, AL.CHI.MI.A. S.R.L., Moria Group, 35020 Ponte San Nicolò, Italy; jtothova@alchimiasrl.com (J.D.T.); 2 Department of Biology, University of Padova, 35131 Padova, Italy; 3 Fondazione Banca Degli Occhi del Veneto ETS (FBOV), 30174 Venice, Italy; 4 Studium Patavinum, University of Padova, 35122 Padova, Italy; 5 Quality Control, AL.CHI.MI.A. S.R.L., Moria Group, 35020 Ponte San Nicolò, Italy

**Keywords:** 3D *in vitro* model, Air–Liquid Interface, OECD Test Guideline 492, *in vitro* eye irritation testing, 3Rs principle

## Abstract

For testing ocular irritation, 3D corneal models mimicking the corneal epithelium are considered reliable eye irritation tests and are detailed in regulatory guideline OECD Test Guideline (TG) 492. The aim of the present study was to develop and validate a Reconstructed human Cornea-like Epithelium (RhCE) *in vitro* irritation test method for ophthalmic medical devices according to OECD TG 492. Immortalized Human Primary Corneal Epithelium Cells (IM-HCEpiCs) were cultured on microporous inserts and exposed to an Air–Liquid Interface (ALI). Morphology was examined using standard (immuno-) histological techniques. Viability was quantified with MTT assay. Barrier integrity and function were monitored by trans-epithelial electrical resistance (TEER) and determination of IC_50_ using MTT assay. Reproducibility was evaluated by calculating the inter-batch coefficient of variation (CV %) of the absorbance values of negative control-treated RhCE model replicates by MTT assay. Technical proficiency was verified using reference chemicals. Irritancy of ophthalmic medical devices was assessed. IM-HCEpiCs developed an epithelium-like barrier under the ALI. TEER increased after ALI introduction, and the obtained IC_50_ value showed concordance with the guideline’s reference ranges. The developed RhCE test method demonstrated technical proficiency and correctly identified medical devices as non-irritants. A novel RhCE model was developed and validated according to OECD TG 492.

## 1. Introduction

Medical devices designed to contact human ocular tissues must be free of substances that could cause irritation or eye damage, requiring thorough testing to ensure safety [1]. Accordingly, developers of ophthalmic medical devices intended to come in contact with a patient’s tissue are required by regulatory authorities to perform a biological risk evaluation, testing their safety [2]. An essential aspect of biocompatibility testing involves the evaluation of potential irritation that may be caused upon interaction of medical devices with eyes, where the cornea serves as a primary barrier.

In humans, the cornea is the outermost part of the eye [3]. It is an avascular and transparent tissue that serves as the first site of contact with the external environment and as a protective barrier from environmental insults [4]. It consists of five layers: moving from anterior to posterior, there is the epithelium, which protects the eye and is in contact with the external environment (air); the stroma, the thickest layer, providing structure; the endothelium, which maintains corneal transparency by regulating fluid entry and exit in the stroma; and the Bowman membrane and Descemet membrane, which separate the epithelium from the stroma and the stroma from the endothelium, respectively [5]. Routinely, the cornea comes in contact with different kinds of substances that may elicit eye irritation or damage [6].

Eye irritation refers to the occurrence of reversible changes in the eye after exposure to an irritating substance [7]. Assessment of ocular irritancy usually demands animal testing [8]. These examinations are comprehensive as they can capture a broad range of responses altogether, such as opacity, irritation, conjunctivital discharge, inflammation, immune reactions, and edema [9]. However, these studies also have certain limitations, including being time-consuming, costly, variable in outcomes [6] and subjected to ethical considerations. Consequently, a variety of initiatives has been implemented to integrate alternative testing methods into regulatory practices [10] based on Russel and Burch’s 3Rs principle, *i.e.*, Reduction, Refinement, and Replacement of animals in safety testing [11].

These alternative approaches include testing methods that rely on the use of *ex vivo* and *in vitro* models [2]. *Ex vivo* models are tissues sourced from animals such as chicken [12] and bovine [13] models, while *in vitro* testing methods involve the use of monolayer cultures [12] or a human-based three-dimensional (3D) model cultured on synthetic porous inserts [13] that are developed using the air–liquid interface (ALI) cell culture method [14]. The latter offers the advantage of more accurately representing morphological features of the native tissue than monolayer cultures, thereby enabling evaluation of a broader range of endpoints, such as changes in barrier function [15] and morphology, following chemical exposure [6].

So far, for testing ocular irritation, 3D corneal models mimicking the corneal epithelium are considered validated eye irritation tests and are well detailed in OECD TG 492 [7]. The rationale behind the guideline is the assumption that all chemicals inducing serious eye damage or eye irritation will induce cytotoxicity in the corneal epithelial cells regardless of the physicochemical processes involved in cell death. In general, the guideline identifies the use of Reconstructed human Cornea-like Epithelium (RhCE) models grown on porous synthetic inserts to identify chemicals based on the United Nations Globally Harmonized (UN GHS) classification system [16]. According to this classification system, chemicals causing serious eye damage are classified as “Category 1,” while those that lead to eye irritation are classified as “Category 2.” On the other hand, chemicals that do not fall into either of these categories are labelled as UN GHS “No category,” indicating they do not require any classification for eye irritation or serious eye damage [7,16].

The RhCE models described in OECD TG 492 identify chemicals falling into the UN GHS “No category” (non-irritant). They are not provisioned in the guideline for the categorization of chemicals falling into Category 1 and Category 2, which would necessitate further testing depending on regulatory requirements [8,12,13,17,18].

It is important to highlight that in vitro irritation testing methods that employ human-based 3D models cannot completely replace animal testing, which can capture the full range of serious eye damage/eye irritation responses for different chemical classes. However, they can be used in combination with other alternative testing methods within a tiered testing strategy, such as bottom-up and top-down approaches, to substantially decrease reliance on animals [7,10]. Some 3D *in vitro* corneal epithelium models have been developed and met regulatory validation standards. These models are listed in OECD TG 492. Consequently, for organizations conducting routine irritation testing, purchasing these models is often not a cost-effective solution. Moreover, no studies, to our knowledge, have applied OECD TG 492-compliant RhCE models to verify the absence of irritancy of ophthalmic medical devices, particularly those used for the preservation of donor human corneas. Therefore, the aim of the present study was to develop and validate a novel RhCE model using SV40-transduced human corneal epithelial cells (IM-HCEpiCs) and to employ it as a reproducible, time- and cost-effective tool for an *in vitro* irritation test method to evaluate potential irritancy of ophthalmic medical devices in accordance with OECD TG 492.

## 2. Materials and Methods

### 2.1. Cell Culture

Immortalized Human Corneal Epithelial Cells (IM-HCEpiCs, Innoprot, Derio, Spain, Ref: P10871-IM) were cultured in high-glucose DMEM (Merck Sigma, St. Louis, MO, USA, Ref: D6429) supplemented with 10% heat-inactivated FBS (Gibco, Auckland, New Zealand, Ref: 10094-148), 5 µg/mL human recombinant insulin (Merck Life Sciences, St. Louis, MO, USA, Ref: 91077C), 0.5% DMSO (CRYO-ON, AL.CHI.MIA SRL, Ponte San Nicolò, Italy, Ref: CRN 002-00), 10 ng/mL epidermal growth factor (EGF) (Merck-Sigma, St. Louis, MO, USA, Ref: E9644), penicillin–streptomycin (Merck-Sigma, St. Louis, MO, USA, Ref: PO781) at a final concentration of 100 U/mL penicillin and 100 μg/mL streptomycin, and 0.25 μg/mL Amphotericin B (Merck-Sigma, St. Louis, MO, USA, Ref: A9528) in T75 cm^2^ flask (Falcon, Marlboro, NY, USA, Ref: 353136). The flasks were previously coated with 5 mL FNC Coating Mix (Athena ES, Baltimore, MD, USA, Ref: 0407) for 15 min. The cells were maintained in an incubator at standard culture conditions (5% CO_2_, 90–95% relative humidity (RH), 37 °C) and sub-cultured upon reaching 90% confluence.

### 2.2. Development of RhCE Model

A total of 450 μL of cell suspension of IM-HCEpiCs were seeded onto FNC-coated PET clear hanging inserts (pore size of 0.4 μm and membrane growth area 0.3 cm^2^, CellQART^®^, Northeim, Germany, Ref: 9320412) at a final density of 0.6 × 10^5^ cells/mL per insert integrated in a 24-well plate (Corning, Corning, NY, USA, Ref: 3527). Culture medium (800 μL) was added to the basolateral side. The cells were allowed to grow in a liquid–liquid interface (LLI) under standard culture conditions until they reached full confluence (approximately 100% coverage of the growth area), typically after 3–4 days from seeding. Subsequently, cells were exposed to an air–liquid interface (ALI) by completely removing the culture medium from the apical side and cultured for 4–6 additional days, bringing the total culture duration to 8–9 days. Cells cultured for 3 days in LLI followed by 5–6 days in ALI are designated as ALI 3 (8–9), whereas cells cultured for 4 days in LLI followed by 4–5 days in ALI are referred to as ALI 4 (8–9). These culture conditions were set to accommodate variations in procedures at companies, considering time-dependent factors and potential real-world conditions that may influence the day of conducting irritation testing. The medium in the basolateral side was changed daily after transitioning to ALI.

### 2.3. Viability and Reproducibility of the RhCE Model

The viability of the RhCE model was evaluated using a standardized MTT colorimetric assay. The tetrazolium salt MTT (3-(4,5-dimethylthiazol-2-yl)-2,5-diphenyltetrazolium bromide) acts as an indicator of cellular viability because it measures mitochondrial succinate dehydrogenase activity; disruptions in enzyme function and changes in cellular energy production can therefore be inferred from the resulting viability values. MTT is able to cross the plasma membrane as well as the mitochondrial inner membrane, where the dye is reduced to purple formazan by mitochondrial enzymes of viable cells. The amount of formazan is then quantified by measuring absorbance at 570 nm.

MTT was purchased from Merck-Sigma, St. Louis, MO, USA, Ref: M2128-5G. All procedural steps were carried out under aseptic conditions within a laminar flow hood.

RhCE model replicates exposed to ALI (n = 9) served as samples for viability assessment. Additionally, RhCE model replicates (n = 13) treated with 50 μL of Ca^2+^- and Mg^2+^-free DPBS (Merck- Sigma, St. Louis, MO, USA, Ref: D8537-500ML) were considered negative controls and those (n = 10) treated with 50 μL of pure methyl acetate (Merck-Sigma, St. Louis, MO, USA, Ref: 186325) were included as positive controls. All RhCE model replicates exposed to different conditions were then incubated at standard culture conditions for 30 min, followed by washing with 150 μL of Ca^2+^- and Mg^2+^-free DPBS, and transferred into a 24-well plate containing 0.4 mL of 1 mg/mL MTT solution. Then, they were incubated for 3 h in a CO_2_ incubator at standard culture conditions to allow metabolically active cells of the model to reduce MTT dye (supplied from the basolateral side of the inserts) to purple formazan crystals.

Formazan crystals were extracted by adding 300 μL of 2-propanol (Merck-Sigma, St. Louis, MO, USA, Cas No. 67-63-0, Ref: 3359-2.5L-M) to the apical side of the model by overnight incubation at 2–8 °C in the dark. To prevent evaporation of 2-propanol, plates were sealed with Parafilm^® ^(Amcor, Zurich, Switzerland, Ref: HS234526B).

On the following day, the extraction solution containing formazan crystals was transferred from each RhCE model sample into a 96-well plate (two wells per RhCE model condition, each containing 100 μL) (Corning, Corning, NY, USA, Ref: 3599). For viability quantification, optical density (OD) was measured at 570 nm with a spectrophotometer (BioTek Instruments, Winooski, VT, USA, Ref: ELx800) and RhCE viability (%) was calculated relative to the negative control using the formula described in Equation (1).

(1) $$Cell\;Viability\left[\%\right]=\frac{{OD}_{sample}^{570nm}-{OD}_{blank}^{570nm}}{{OD}_{negative\;control}^{570nm}-{OD}_{blank}^{570nm}}$$

where OD^570nm^_blank_, OD^570nm^_sample_ and OD^570nm^_negative control_ correspond to optical density at 570 nm of neat 2-propanol, the RhCE model treated with samples and the RhCE model treated with the negative control, respectively. Viability data were obtained from four different RhCE preparation batches. Reproducibility of the RhCE model was evaluated by calculating the coefficient of variation (CV %) of the optical density measured at 570 nm for the negative control-treated RhCE model. Mean OD values from at least two RhCE model replicates per experiment were analyzed across 18 independent experimental runs.

### 2.4. Barrier Integrity Assessment

RhCE barrier integrity was assessed by measuring trans-epithelial electrical resistance (TEER), which measures the electrical current (ion flow) across a growing cellular barrier and quantifies the tightness of cell junctions [18]. TEER was recorded at different timepoints (24 h before ALI introduction, day of ALI introduction and days 1, 3, 4 and 6 after ALI introduction) during the culture period with an STX4 electrode (World Precision instruments, Sarasota, FL, USA, Ref: EVM-EL-03-03-01) and EVOM™ Manual Meter (World Precision Instruments, Sarasota, FL, USA, Ref: EVM-MT-03-01) following the manufacturer’s instructions. Electrodes were disinfected by immersion in 70% isopropyl alcohol (Klercide, Northwich, Cheshire, UK, Ref: 3078790) for 10 min before use. For measurements, 450 μL and 800 μL of conductive solution (Ca^2+^ and Mg^2+^ free-DPBS) were added to the apical and basolateral sides of the blank inserts, respectively. The instrument was calibrated, equilibrated and blank measurements were recorded on inserts with conductive solution devoid of cells to establish baseline resistance values. For each measurement day, at least n ≥ 20 RhCE model replicates were used to measure TEER values. Cells seeded at the same density on PET inserts and not exposed to ALI were used as the control group. Unit area resistance was calculated using the formula described in Equation (2).

(2) $$Resistance\;of\;unit\;area\left(\Omega .{cm}^{2}\right)=Resistance\left(\Omega \right)\cdot Effective\;membrane\;area\;\left({cm}^{2}\right)$$

The mean and SD of the TEER recordings measured on each day were calculated.

### 2.5. Histology and Immunohistochemistry Examinations

For morphological evaluation of the RhCE model, n = 5 RhCE model replicates cultured for 3 days in LLI and 5 days in ALI [ALI 3(8)] and n = 4 cultured for 4 days in LLI and 4 days in ALI [ALI 4(8)] were fixed in 10% formalin (Sigma Aldrich, St. Louis, MO, USA, Ref. No.HT501128-4 mL) for 45 min and exposed to increasing sucrose (Millipore, Burlington, MA, USA, 8510-500gm) concentrations (10% for 3 h, followed by 15% for 3 h and finally 30% overnight). RhCE model replicates on PET membranes were carefully separated from the culture insert walls using a scalpel, embedded in Tissue-Tek Optimal Cutting Temperature Compound (Sakura Finetek, Torrance, CA, USA, Ref. 4583) and frozen on nitrogen fumes.

Ten (10) µm sections were cut using a Leica CM 1510-1 cryostat and stained with ST Infinity Hematoxylin and Eosin (H&E) Staining System (Leica, Nussloch, Germany, Ref: 3801698) according to the manufacturer’s instructions. Briefly, the sections were rehydrated through passages in 100% reagent alcohol (Leica, Ref. 3803686), 80% reagent alcohol and water, and incubated for 30 s in ST HemaLast. The samples were then stained for 3 min in ST Hematoxylin, followed by a wash in water, incubation in an ST Differentiator for 45 s, a wash and incubation in ST Bluing agent for 1 min. Finally, the slides were passed in 80% reagent alcohol, stained with ST Eosin for 50 s, dehydrated in 100% reagent alcohol, cleared in xylene (Leica, Ref. 3803665EG) and mounted with CV Ultra mounting medium for cover slipping slides (Leica, Ref: 14070937891).

For immunofluorescence characterization, 5 µm frozen RhCE sections were permeabilized in 0.5% Triton X-100 (Merck-Sigma, St. Louis, MO, USA, Ref: T8787) in PBS for 20 min and incubated in 5% bovine serum albumin (BSA, Merck, Ref:A7906) for 30 min. Unconjugated antibodies (mouse anti-Cytokeratin 3 clone AE5, Ref: MA5-46556, rabbit-anti-Claudin 3, Ref: 34-1700, mouse anti-Occludin, Ref: 33-1500, ThermoFisher Scientific, Waltham, MA, USA) were used at 1:100 dilution in 1% BSA overnight at 4 °C, followed by washing with PBS and incubation with Donkey anti-Mouse IgG (H + L) Highly Cross-Adsorbed Secondary Antibodies, Alexa Fluor™ Plus 488, ThermoFisher Scientific, Waltham, MA, USA, Ref: A32766; anti-Mouse IgG (H + L) Highly Cross-Adsorbed Secondary Antibody, Alexa Fluor™ Plus 594, ThermoFisher Scientific, Waltham, MA, USA, Ref: A32744; and anti-Rabbit IgG (H + L) Highly Cross-Adsorbed Secondary Antibody, Alexa Fluor™ Plus 488 ThermoFisher Scientific, Waltham, MA, USA, Ref: A32790TR at 1:200 dilution in 1% BSA for 2 h at 37 °C. Anti-ZO-1 (Zona Occludens-1) Monoclonal Antibody, Ref: ZO1-1A12, Alexa Fluor™ 488, ThermoFisher Scientific, Waltham, MA, USA, was incubated at 1:100 dilution in 1% BSA for 2 h at 37 °C. Finally, the slides were mounted with DAPI-Fluoromount-G™ mounting medium containing 4-6 diamidino-2-phenylindole (DAPI) for nucleus counterstaining (Electron Microscopy Sciences, Hatfield, PA, USA, Ref: 17984-24). All pictures were taken using a Nikon Eclipse Ti microscope (Nikon, Tokyo, Japan) with a 40× objective. The thickness of the RhCE model was quantified using FIJI software version 20250529-2217 [2]. For each RhCE model replicate, n ≥ 3 slices were prepared. Within each slice, thickness measurements across at least three distinct regions were obtained and an average thickness value was calculated for each slice. Means and SD were calculated across all representative slices for each RhCE model sample. Subsequently, the overall average thickness across the RhCE model was determined.

### 2.6. Barrier Function Assessment

Barrier functionality of the RhCE model was assessed through the determination of its resistance to the permeation of sodium dodecyl sulfate (SDS), an established cytotoxic benchmark substance, followed by calculating its half maximal inhibitory concentration (IC_50_). IC_50_ is the concentration of SDS that reduces 50% viability of the RhCE model at a fixed exposure time (30 min) [13].

IC_50_ for the RhCE model was assessed across eight independent experiments. RhCE model replicates at culture condition ALI 3-4 (8–9) were employed for IC_50_ estimation. Fifty µL of different concentrations (1, 2 and 3 mg/mL in Ca^2+^- and Mg^2+^-free DPBS) of SDS (Sigma-Aldrich, Ref. No. 05030, St. Louis, MO, USA) were applied apically to n ≥ 3 RhCE model replicates for each concentration per experiment. Post application, RhCE model replicates were maintained at standard cultured conditions in an incubator for 30 min. Within each individual experiment, % viability of the RhCE model after exposure to scalar SDS concentrations was evaluated with MTT assay, as described in Section 2.3. IC_50_ was obtained (see Section 2.9) by considering data from 6 independent experimental runs.

### 2.7. Technical Proficiency of RhCE Model-Based In Vitro Irritation Test

To assess the technical proficiency of the *in vitro* irritation test utilizing the described RhCE model, n = 15 chemicals listed in OECD TG 492 were purchased from Merck-Sigma, St. Louis, MO, USA and employed as reference standards for conducting irritation assessments: methyl thioglycolate (Cas No. 2365-48-2, Ref:108995); hydroxyethyl acrylate (Cas No.818-61-1, Ref: 292818); 2,5-dimethyl-2,5-hexanediol (Cas No.110-03-2, Ref: 143618); sodium oxalate (Cas No. 62-76-0, Ref: 223433); 2,4,11,13Tetraazatetradecanediimidamide, N,N″-bis(4chlorophenyl)-3,12diimino-, di-D-gluconate (20%, aqueous) (chlorhexidine digluconate, 20%, aqueous solution) (Cas No. 18472-51-0, Ref: C9394); sodium benzoate (Cas No. 532-32-1, Ref: 09169); diethyl toluamide (Cas No. 134-62-3, Ref: D100951); 2,2-dimethyl-3-methylenebicyclo[2.2.1]heptane (Camphene, Cas No. 79-92-5, Ref: 456055); 1-ethyl-3-methylimidazolium ethyl sulfate (Cas No. 342573-755, Ref: 51682); dicaprylyl ether (Cas No. 629-82-3, Ref: 249599); piperonyl butoxide (Cas No. 51-03-6, Ref: 291102); castor oil (Cas No. 61788-85-0, Ref: 07076); 1-(4-chlorophenyl)-3-(3,4-dichlorophenyl)urea (Cas No. 101-20-2, Ref: 105937); 2,2′-methylene-bis(6-(2H-benzotriazol-2-yl)-4-(1,1,3,3-tetramethylbutyl)phenol) (Cas No. 103597-45-1, Ref: 407941); potassium tetrafluoroborate (Cas No. 14075-53-7, Ref: 278955). This list includes UN GHS-classified chemicals covering different physical states and organic functional groups, and have already been tested by *in vivo* rabbit eye test [13].

Protocols for testing chemicals on the RhCE model accounted for the physical state of the test chemicals and varied in dose amount (50 μL for liquid chemicals, 20 mg for solid chemicals, weighed in 1.5 mL Eppendorf^®^ Safe-Lock microtubes, Hamburg, Germany, Ref: EP0030123328) and exposure duration (30 min for liquids, 24 h for solids), per OECD TG 492. To prevent cross-contamination of volatile chemicals, treated and control samples were incubated in separate 24-well plates during the experiment. Some chemicals needed additional processing steps due to their chemical–physical properties, which could potentially interfere with the assay as follows:

(i). Camphene, which was inherently sticky and volatile, was solubilized in perfluorodecalin (HPF10, AL.CHI.MI.A SRL, Ponte San Nicolò, Italy, Ref: HPF 003-00) containing 4% *v*/*v* ethanol (Carlo Erba, Milan, Italy, Ref: 414608) prior to application. This approach was adopted to ensure its uniform application onto the model’s surface. Nevertheless, the duration of exposure to camphene was maintained at 24 h, as pure camphene existed in solid form at standard environmental conditions. RhCE model replicates treated with perfluoro-decalin + 4% ethanol without camphene were added as additional controls in the experiment. Control RhCE replicates were incubated separately from those treated with camphene to prevent exposure to camphene vapors. Percent viability of camphene-treated RhCE model replicates was calculated relative to perfluoro-decalin + 4% ethanol-treated RhCE model replicates.
(ii). Before testing chemicals on the RhCE model, their potential to directly reduce MTT dye was assessed. Solid chemicals (50 mg) and liquid chemicals (50 µL) were added to 0.4 mL of a 1 mg/mL MTT solution and incubated for 3 h. A chemical was considered a direct MTT reducer if the solution developed a purple color post incubation. Methyl thioglycolate and piperonyl butoxide exhibited direct MTT dye reduction activity; therefore, additional controls were included along with conventional negative and positive controls by exposing RhCE model replicates to −80 °C for 30 min in two cycles (RhCE^freeze-killed^). Direct MTT-reducer chemicals were then applied concurrently to RhCE^freeze-killed^ controls to identify and quantify any non-specific reduction of the MTT dye by the chemical itself, independent of the cell viability within the RhCE model system. Percent viability was determined using Equation (3).
(3) $$\begin{array}{c} {Cell\;Viability}_{\left(DirectMTT-reducers\right)}\left[\%\right]\\ \;\;\;\;\;\;\;\;\;\;\;\;\;\;={Cell\;Viability}_{teated\;with\;direct\;MTT\;reducers}^{live\;RhCE}\\ \;\;\;\;\;\;\;\;\;\;\;\;\;\;-{Cell\;Viability}_{treated\;with\;direct\;MTT\;reducers}^{freeze-killed\;RhCE} \end{array}$$
(iii). Castor oil, a highly viscous chemical, was pre-warmed to 37 °C for 20 min to ensure full liquefaction and facilitate application. Post exposure on the RhCE model, it was removed using a cannula-27G injection, followed by three gentle rinses with 0.3% Tween 80 (Merck-Sigma, St. Louis, MO, USA, Ref: P4780) in DPBS to remove residues. Negative and positive controls received identical washing treatment to ensure experimental consistency.

For testing each chemical on the RhCE model, at least two (n ≥ 2) replicates were used per individual experiment, across at least 3 independent experiments using different RhCE batch preparations. RhCE model replicates post-chemical treatment were incubated at standard culture conditions. The viability of chemical-treated RhCE model replicates was calculated relative to the negative control with MTT assay, as described in Section 2.3. A viability cut-off value of >60% was set as the threshold to classify chemicals as non-irritants. For chemicals reducing viability below the set thresholds, no conclusive prediction could be made. They were classified into the “No prediction could be made” category, indicating the need for further testing to determine their eye hazard potential.

### 2.8. In Vitro Irritation Test on Medical Devices

The RhCE-based *in vitro* irritation model was employed to evaluate the irritancy of the following medical devices from AL.CHI.MI.A. S.R.L (Moria group, Ponte San Nicolò, Italy): OCIGEL (Ref: OCI 001; OCI 002), eyeDRO (Ref: EDO 001), PSS-L (Ref: GRS 003-00), TISSUE-C (Ref: TIS 001-00), CARRY-C (Ref: CAR 001-00), Eusol-C (Ref: CSM 001-00), KERASAVE (Ref: KER 002-00) and XTRA4 (Ref: EXT4 001-01).

To assess any potential color interference (*i.e.*, absorption of light in the same wavelength range of formazan dye) of medical devices, a preliminary test was performed in which 100 µL of the medical device was added to 900 µL of distilled water and incubated for 30 min at room temperature to observe whether the solution developed any strong coloration. Optical density was measured at 570 ± 20 nm. OD570 ± 20 nm values exceeding 0.08 were set as the threshold for deeming color interference, as established by validated reference methods 1 and 2 (VRM1 and VRM2) outlined in OECD TG 492 [13]. Medical devices were also evaluated for direct MTT-reducing activity using the same protocol applied to test the chemicals described in Section 2.7. As all tested medical devices were in liquid form, they were applied on the RhCE model following the protocol described for liquid chemicals in Section 2.7. Mean % viability was evaluated with MTT assay, as described in Section 2.3. At least two (n ≥ 2) RhCE model replicates were used per individual. Data were obtained from n ≥ 3 independent experiments employing different RhCE batch preparations.

### 2.9. Data Analysis and Statistics

Data visualization, including scatter plots for correlation analysis and bar graphs for group comparisons as well as data and statistical analyses, was performed using Excel Microsoft Office 2019 software (Microsoft Corp., Redmond, WA, USA) with the plugin Real Statistics Pack (www.real-statistics.com, 2013). All data were assessed for normality and homogeneity of variances using the Shapiro–Wilk test and Levene’s test, respectively. Unless otherwise specified, data were expressed as mean ± standard deviation (SD), and statistical significance was defined as *p* < 0.05.

For viability experiments (refer to Section 2.3), statistical comparisons between experimental and control groups were conducted using the Kruskal–Wallis test. Subsequent pairwise comparisons were performed with the Mann–Whitney U test. To account for multiple testing, *p*-values were adjusted using Bonferroni correction. Differences in thickness of RhCE model preparations at culture conditions between ALI 3(8) and ALI 4(8) (see Section 2.5) were assessed using the Mann–Whitney U test for two independent groups.

For TEER measurements (refer to Section 2.4), differences in mean TEER values across various days within the group were evaluated using the Kruskal–Wallis test. For pairwise comparisons between TEER measurements recorded on days post ALI and those obtained 24 h before ALI introduction, Dunn’s post hoc test was performed. Differences in TEER values between the RhCE model and the control group on day 10 were assessed using the Mann–Whitney U test for two independent groups.

IC_50_ estimation was performed by constructing a calibration curve plotting SDS concentration ([SDS], expressed in mg/mL) on the abscissa versus the % viability of the RhCE model (calculated relative to the negative control) on the ordinate. The obtained calibration curve was fitted with a linear equation (*y* = mx + q, where y = % viability, *m* = slope of the calibration curve, *x* = [SDS], *q* = the y-intercept of the calibration curve), and the coefficient of determination (*R*^2^) was calculated to assess the goodness of fit of the regression model. The IC_50_ was then estimated from the linear equation as the SDS concentration (*x*) required viability to be reduced by 50% (*y* = 0.5). Comparisons between RhCE model replicates treated with different SDS concentrations were performed using the Kruskal–Wallis test, followed by Dunn’s test for post hoc pairwise comparisons. To evaluate the level of agreement between the RhCE irritation test method used in this study and the reference methods described in OECD TG 492, Cohen’s *κ* statistic was calculated. Results from the test methods were categorized into binary outcomes (*e.g.*, no prediction vs. non-irritant). A 2 × 2 contingency table was created to summarize concordant and discordant results between the methods. The *κ* coefficient was computed to quantify agreement beyond that expected by chance. The 95% confidence intervals (95% CI) for *κ* were calculated using the standard error-based asymptotic method.

## 3. Results

### 3.1. RhCE Model Viability and Reproducibility

**Figure 1:**
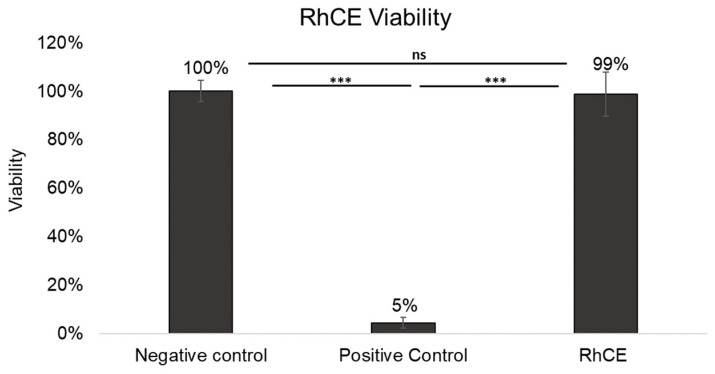
Average % viability of RhCE model relative to negative control on 8^th^ day of total culture duration compared across n = 4 different experiments. Negative control: RhCE model (n = 13) treated with 50 µL of Ca^2+^- and Mg^2+^-free DPBS, ensuring a baseline viability of 100%; positive control: RhCE model treated with 50 µL neat methyl acetate (n = 10) and untreated RhCE model (n = 7). *** *p* < 0.001; ns = not significant (Kruskal–Wallis test followed by pairwise Mann–Whitney U test comparisons).

The RhCE model displayed OD^570nm^ 1.31 ± 0.26, consistently falling within acceptability ranges for negative control values established by VRMs (VRM1: >0.8 to <2.8; VRM2: >1.0 to ≤2.5) according to OECD TG 492. The average % viability of the RhCE model calculated using the OD^570nm^ (see Section 2.3) was statistically comparable (*p* = 0.1537) to the negative controls, as shown in Figure 1. In contrast, the RhCE model treated with methyl acetate (positive control) exhibited a drastic reduction in viability (Figure 1). The negative control OD^570nm^ (1.41 ± 0.19) displayed a CV % of 13.3%.

### 3.2. Histological and Immunohistochemical Evaluation of the Novel RhCE Model

**Figure 2:**
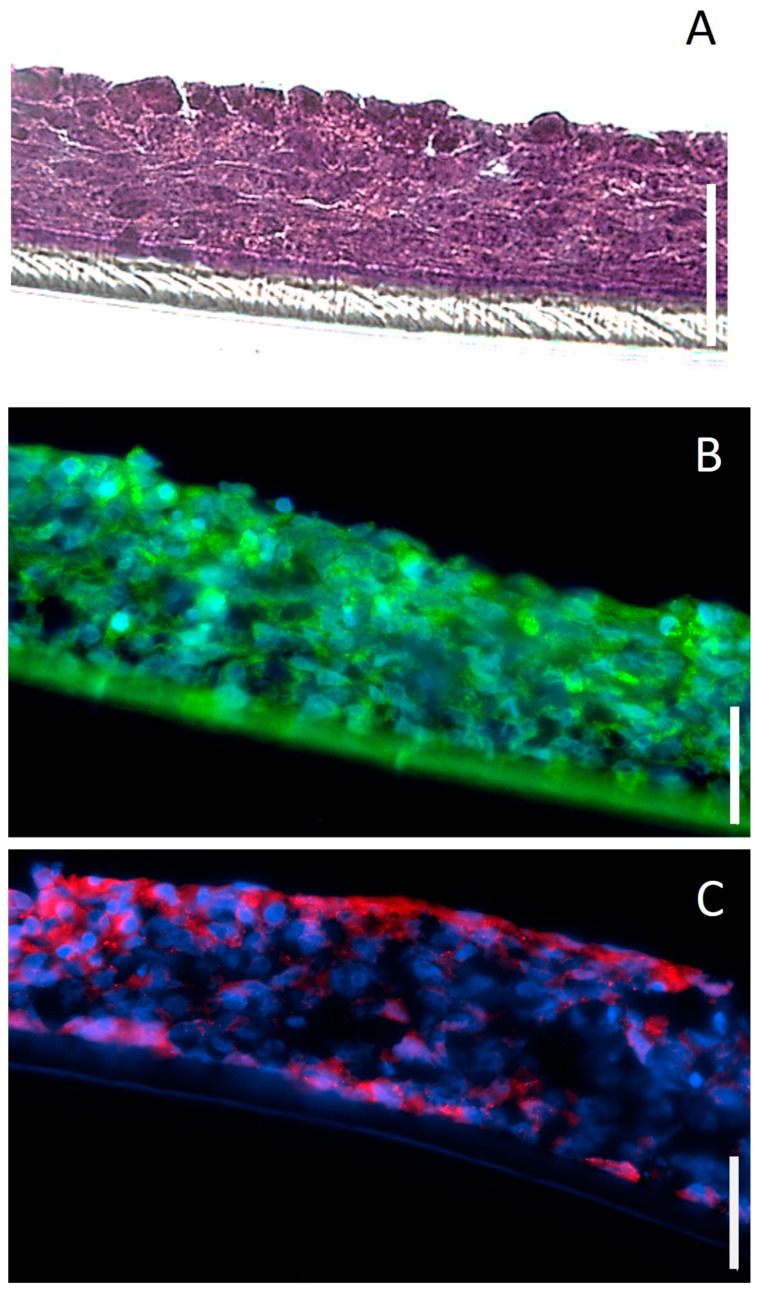
Cross-sectional images of RhCE tissue model cultured for a total of 8 days. (**A**) H&E-stained section (10 μm thickness). (**B**) Immunohistochemical staining for CK-3 (green) and (**C**) ZO-1 (red) in 5 μm thick sections. Nuclei were stained with DAPI (blue). Scale bar: 50 μm.

The RhCE model exhibited stratified cellular organization (Figure 2A), with five to six cellular layers and a non-keratinized surface. Its thickness was observed to vary significantly by the day of ALI exposure. The RhCE model cultured under ALI 3(8) conditions exhibited a thickness of 66.0 ± 12.6 μm compared to 44.9 ± 6.5 μm under ALI 4(8) conditions (*p* < 0.001). Immunohistological analysis indicated that the RhCE model expressed corneal epithelium-associated markers such as CK-3 and ZO-1 (Figure 2B,C). CK-3 was observed to be consistently expressed throughout the entire thickness of the model (Figure 2B), while ZO-1 expression was localized on the apical cellular layers (Figure 2C). To evidence the interaction between tight junctions and the cytoskeleton, we confirmed the expression of Claudin-3 and Occludin (Supplementary Figure S1).

### 3.3. Barrier Integrity

**Figure 3:**
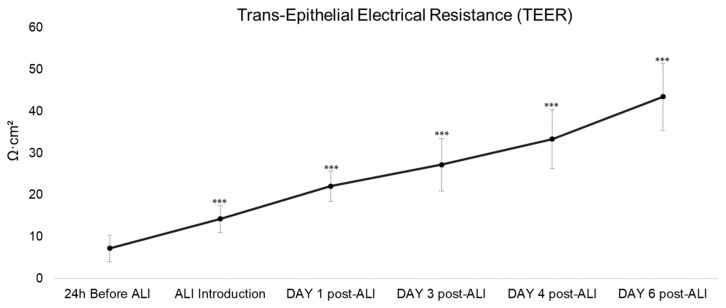
Monitoring of barrier integrity of the RhCE model by determination of TEER (average ± standard deviation, expressed in Ω·cm^2^) at different time points during culture up to 6 days post ALI introduction compared to 24 h before ALI. For each day, at least n ≥ 10 RhCE model replicates were included, *** *p* < 0.001 (Kruskal–Wallis test followed by Dunn’s multiple comparisons).

As shown in Figure 3, the RhCE model displayed a progressive increase in TEER, from 7.2 ± 3.1 Ω·cm^2^ when ALI was introduced, reaching an average of 43.5 ± 8.1 Ω·cm^2^ on the final day of the culture duration (day 10, with ALI introduced on day 3–4). The increase was statistically significant compared to the TEER recorded before ALI introduction (*p* < 0.001) (Figure 3). A similar increasing trend was observed in the control group; however, by day 10, TEER values averaged around 22.0 ± 4.8 Ω·cm^2^, significantly lower than those of the RhCE model (*p* < 0.001) (Supplementary Figure S2).

### 3.4. Barrier Function

**Figure 4:**
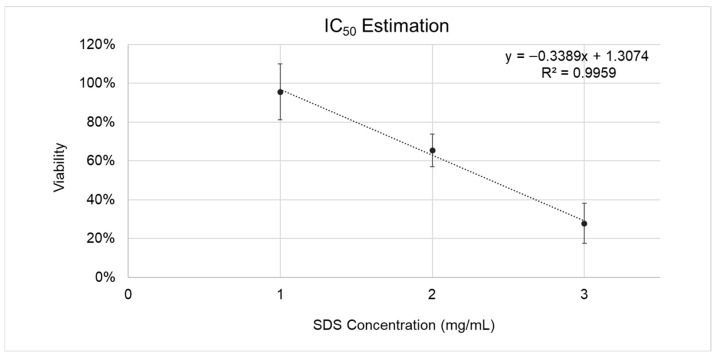
Calibration curve illustrating IC_50_ estimation to assess the barrier function of the developed RhCE model (airlifted on days 3–4 and subsequently cultured for an additional 5 days) with MTT assay. Percent viability (%) calculated relative to negative control (RhCE model treated with 50 µL of Ca^2+^- and Mg^2+^-free DPBS) values were converted to fractions (0–1) for the purpose of fitting the linear regression equation and calculating IC_50_. IC_50_ was determined as the SDS concentration producing 50% reduction in viability (*y* = 0.5). The curve represents SDS concentration (presented as mg/mL) vs % viability for each concentration of SDS. From n = 14 experiments, with n = 3 RhCE model replicates per condition for each experiment. Calibration curve equations and *R*^2^ are presented on the graphs.

Exposure to increasing SDS concentrations induced a dose-dependent decrease in RhCE viability. The curve fitting resulted in a linear equation (*y* = −0.3389*x* + 1.3074) indicating an inverse correlation (slope: −0.3389; *R*^2^: 0.9959) between RhCE viability and SDS concentration (Figure 4). Using this equation, the IC_50_ of the RhCE model was estimated to be 2.4 mg/mL, which fell within the accepted IC_50_ QC batch release acceptance range of VRM2 (1–3.2 mg/mL), a commercially available *in vitro* model for ocular irritation testing.

### 3.5. Technical Proficiency of RhCE Model

Table 1 presents the mean RhCE % viability of the RhCE model following exposure to 15 proficiency chemicals enlisted in OECD TG 492. The *in vitro* eye irritation test employing the described RhCE model correctly predicted six out of seven non-irritants as the model’s viability was maintained above 60% threshold post-exposure. These results aligned with OECD TG 492 predictions for these chemicals. However, 1-ethyl-3-methylimidazolium ethyl sulfate, classified as a non-irritant in the guideline, elicited cytotoxicity (RhCE viability: 12.31 ± 8.36%) in the RhCE model and hence could not be classified as a non-irritant. All chemicals from the OECD “No prediction can be made” category were correctly identified by RhCE irritation test, reducing the model’s viability to below the 60% threshold. Agreement between the RhCE test and other reference test methods mentioned in OECD TG 492 was observed to be high, with a Cohen’s *κ* of 0.86 (95%CI: 0.61–1.00).

### 3.6. In Vitro Irritation Test of Medical Devices

**Figure 5:**
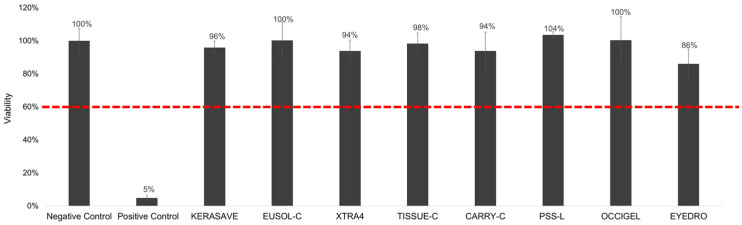
Average *%* viability of the RhCE model, relative to the negative control, following exposure to medical devices showing no irritancy . For each experiment, at least two RhCE model replicates per each treatment (control and medical devices) were included, with data pooled from at least three different seeding preparations. Negative control: RhCE model receiving 50 µL of Ca^2+^- and Mg^2+^-free DPBS; positive control: RhCE model treated with 50 µL neat methyl acetate.

All medical devices showed no significant color interference at 570 ± 20 nm (OD^570nm^ < 0.08) nor direct MTT reduction activity (Supplementary Table S1). The RhCE model maintained viability above the 60% threshold following exposure to all the medical devices evaluated (Figure 5); consequently, all devices were classified as non-irritants by the *in vitro* irritation test.

## 4. Discussion

Companies developing and commercializing medical devices must ensure their products do not irritate contacting tissues, generally examined through irritation tests that have traditionally relied on animals [2]. To minimize animal use and reduce costs, regulatory authorities such as the U.S. Food and Drug Administration (FDA) and EU regulatory authorities have encouraged the adoption of alternative testing methods [19,20]. At the regulatory level, OECD TG 492 is a guideline that identifies the use of a Reconstructed human Corneal Epithelium (RhCE) model for identifying non-irritant chemicals [7] based on the United Nations Globally Harmonized (UN GHS) classification system [16]. Taking this guideline as a reference, the primary objective of the present study was to develop and validate a reproducible, time- and cost-effective *in vitro* irritation test method utilizing a novel Reconstructed human Cornea Epithelium (RhCE) model in accordance with the OECD Reconstructed Human Cornea-like Epithelium (RhCE) test method for conducting *in vitro* irritation assessment of ophthalmic medical devices. The culture is initiated from a single epithelial cell population, with an air–liquid interface (ALI) promoting both stratification and phenotypic differentiation, combining apical exposure to air with basal nutrient and differentiation-promoting supplements and hormone supply through the culture medium. Under these conditions, epithelial cells proliferate, stratify vertically, and progressively differentiate into a polarized, pseudostratified tissue-like structure.

The new RhCE model used in this study was developed using an immortalized human corneal epithelial cell line. Despite it being known that SV40 large T antigen immortalization exerts transcriptomic deviations from native corneal epithelium [21], in many studies, including ours, it has been indicated that immortalized cell lines retain basic epithelial features such as the ability to form a barrier-like structure under an air–liquid interface, express corneal epithelium-related markers, and exhibit barrier function properties and irritation responses comparable to OECD TG 492-compliant RhCE models [22,23]. Additionally, OECD Test Guideline 492 prioritizes functional endpoints over gene expression analysis to ensure standardized, reproducible regulatory testing.

Cells were cultured on culture inserts for 3–4 days under a liquid–liquid interface (LLI) and for 5–6 days under an air–liquid interface (ALI). These culture conditions were designed to accommodate variations in irritation testing procedures at different companies, considering time-dependent factors and potential real-world conditions that may influence the timing and execution of the tests.

Additionally, animal derivates like FBS were used during the culturing of the presented model, due to cost optimization of the laboratory setting adopted during the developmental phase. In future scaling up of the analytical procedure, replacing the FBS in the medium with recombinant growth factors and hormones can help to completely abate dependence of this method on the use of animal-derived reagents.

Subsequently, the model was characterized and validated based on cell viability, morphology, barrier functionality and overall technical performance to evaluate its suitability as a testing model. MTT assay was employed as an indicator of cytotoxicity and, thus, of potential irritancy, based on the OECD TG 492 assumption that “cytotoxicity plays an important, if not the primary, mechanistic role in determining the overall serious eye damage/eye irritation response of a chemical regardless of the physicochemical processes underlying tissue damage.” The developed RhCE model remained viable along the culture duration, with an observed average OD^570nm^ of 1.41 ± 0.19 that fell within acceptability ranges for negative control OD values set by the VRM1 and VRM2 test methods in OECD TG 492. These results indicate comparable metabolic activity in the RhCE model relative to the referenced methods. Additionally, the observed CV*%* of 13.3*%* (OD^570nm^ of negative control-treated RhCE model replicates) indicates low inter-assay variability and reliable assay performance [24]. Although OECD Test Guideline 492 does not specify exact CV limits, the demonstration of low variability of the negative control across independent experiments may be consistent with the guideline’s requirement to confirm the reproducibility of the RhCE model.

Histological evaluation indicated that the RhCE model replicates cultured under ALI 3(8) conditions developed stratified cellular layers with an average thickness of 66.0 ± 12.6 μm, which is comparable to the thickness of human corneal epithelium, which typically ranges from 50 to 70 μm [25]. Furthermore, immunohistological characterization indicated that the model exhibited strong expression of corneal epithelium differentiation-specific marker cytokeratin CK-3 [26] and tight junction proteins ZO-1, Claudin-3 and Occludin [27]. Stratification of cellular layers, comparable thickness, absence of keratinization and expression of corneal epithelium-related markers, altogether indicate RhCE model’s morphological similarity to native corneal epithelial tissue. The maximum TEER values attained by the RhCE model on day 10, after 6 days of exposure to ALI, was comparatively lower (~40 Ω·cm^2^) than other tissue models, in which TEER exceeded 400 Ω·cm^2^. However, such high TEER values were reached in *in vitro* corneal tissue models developed using different cell lines, passage numbers and culture conditions [25,28,29,30], all of which have been shown to influence TEER measurements [31]. It should be noted that the ultimate role of this novel RhCE model shall be its application in the evaluation of irritancy of chemicals according to OECD TG 492. Therefore, the IC_50_ value of the model, which is the parameter selected in this study to demonstrate barrier functionality, should reasonably align with the ranges reported by other validated reference test methods described in the guideline that utilize a comparable IC_50_ estimation protocol [7,23]. The IC_50_ for the RhCE model described in the present study was estimated to be 2.4 mg/mL, which fell within the range (1.0–3.2 mg/mL) established by VRM2 [23]. This indicates that the RhCE model exhibits a similar level of permeability and resistance to the test compounds in comparison with VRM2. Lastly, technical proficiency of the model was evaluated by testing 15 proficiency chemicals enlisted in OECD TG 492. A threshold of above 60% viability was established to identify non-irritant chemicals comparable to the thresholds established by other reference test methods. The RhCE model presented in the study demonstrated consistent predictive performance by correctly classifying the tested proficiency chemicals, similar to other validated reference methods such as the VRM2 (SkinEthic™) mentioned in OECD TG2 (see Supplementary Table S2), except for 1-ethyl-3-methylimidazolium ethyl sulfate. Contrary to other reference methods that predicted this compound to be a non-irritant, our model classified it into the “No prediction” category, suggesting potential irritancy. This finding is consistent with the UN GHS classification of 1-ethyl-3-methylimidazolium ethyl sulfate reported in PubChem, classifying this chemical as an eye irritant [32]. Overall, the RhCE model showed concordance with OECD TG 492 reference methods, yielding a Cohen’s *κ* = 0.86, indicating its suitability as an irritation test method.

A limitation of this *in vitro* model is that it represents a simplified approximation of the *in vivo* situation and does not incorporate the tear film, mucin layer, or dynamic mechanical stimuli such as blinking. However, it should be noted that the tear film and mucin layer primarily exert a protective function by limiting direct exposure of the corneal epithelium. Their absence therefore represents a conservative, worst-case exposure scenario. Accordingly, while the model does not fully replicate the complexity of the *in vivo* ocular environment, it is considered appropriate and fit for the purpose of screening and hazard identification within its defined applicability domain, in compliance with OECD Test Guideline 492, which is specifically intended for the assessment of eye hazard potential.

All tested ophthalmic medical devices were deemed non-irritant (“No category”) by the RhCE model. These medical devices are representative of a vast array of ophthalmic medical devices coming into contact with the corneal epithelium during diagnostics routine (OCIGEL), anterior chamber surgery (eyeDRO), donor cornea rinsing (PSS-L), donor corneal organ culture (TISSUE-C and CARRY-C) and donor corneal hypothermic storage (Eusol-C, KERASAVE, XTRA4). These findings further confirmed the biocompatibility of the presented medical devices and the irritation tests previously performed on animals according to ISO 10993-1 [33], reinforcing their safety profile for clinical applications.

## 5. Conclusions

In summary, the findings of this study indicate that the novel RhCE model exhibits morphological similarity to native corneal epithelium and concordance with reference methods mentioned by OECD TG 492 in terms of viability, reproducibility and technical proficiency. The model was able to correctly classify all non-irritant proficiency chemicals, with the noted exception of 1-ethyl-3-methylimidazolium ethyl sulfate, which still aligns with its UN GHS data depicted on PubChem. In addition to this, the model’s correct classification of a diverse panel of ophthalmic medical devices as non-irritants further affirms its utility for routine *in vitro* irritation testing. By enabling cost-effective, ethically responsible *in vitro* assessments, the presented model advances the principles of the 3Rs and may offer a viable preliminary *in vitro* irritation test prior to conducting *in vivo* irritation testing of medical devices while maintaining regulatory compliance.

## Figures and Tables

**Table 1:** List of proficiency chemicals enlisted in OECD TG 492 tested on the RhCE model. Percentage (*%*) viability was calculated relative to negative control (see Section 2.3) and comparison of the final results with the classifications predicted by other reference methods in OECD TG 492.

Chemical Name	CAS	Physical State	Viability % (RhCE)	Prediction	OECD Method Prediction
Methylthioglycolate	2365-48-2	L	5.7 ± 2.65	No prediction can be made	No prediction can be made
Hydroxyethyl acrylate	818-61-1	L	5.15 ± 6.16	No prediction can be made	No prediction can be made
2,5-Dimethyl-2,5-hexanediol	110-03-2	S	2.24 ± 2.06	No prediction can be made	No prediction can be made
Sodium oxalate	62-76-0	S	12.76 ± 1.67	No prediction can be made	No prediction can be made
2,4,11,13Tetraazatetradecanediimidamide, N,N″bis(4-chlorophenyl)- 3,12diimino-, di-D- gluconate (20%, aqueous)	18472-51-0	L	7.84 ± 8.20	No prediction can be made	No prediction can be made
Sodium benzoate	532-32-1	S	4.54 ± 2.41	No prediction can be made	No prediction can be made
Diethyl toluamide	134-62-3	L	8.53 ± 2.55	No prediction can be made	No prediction can be made
2,2-Dimethyl-3methylenebicyclo [2.2.1] heptane (CAMPHENE)	79-92-5	S	56.94 ± 5.68	No prediction can be made	No prediction can be made
1-Ethyl-3methylimidazolium ethylsulphate	342573-75-5	L	12.31 ± 8.36	No prediction can be made	Non-irritant
Dicaprylyl ether	629-82-3	L	105.9 ± 10.61	Non-irritant	Non-irritant
Piperonyl butoxide	51-03-6	L	89.93 ± 10.0	Non-irritant	Non-irritant
Castor oil	61788-85-0	Viscous	69.89 ± 6.39	Non-irritant	Non-irritant
1-(4-Chlorophenyl)-3- (3,4dichlorophenyl) urea	101-20-2	S	84.13 ± 10.17	Non-irritant	Non-irritant
2,2′-Methylene-bis-(6- (2Hbenzotriazol-2- yl)- 4(1,1,3,3tetramethylbutyl)- phenol)	103597-451	S	95.76 ± 13.11	Non-irritant	Non-irritant
Potassium tetrafluoroborate	14075-53-7	S	70.89 ± 8.31	Non-irritant	Non-irritant

## Data Availability

The original contributions presented in this study are included in the article/Supplementary Materials. Further inquiries can be directed to the corresponding author.

## Supplementary Materials

The following supporting information can be downloaded at: https://www.mdpi.com/article/10.3390/bioengineering13050545/s1, Figure S1. Cross-sectional images of RhCE tissue model cultured for a total of 8 days. Immuno-histochemical staining for (A) Claudin-3 (green) and (B) Occludin (red) in 5 μm sections. Nuclei were stained with DAPI (blue) Scale bar: 50 μm. Figure S2. Comparison of average TEER (average ± standard deviation, expressed in Ω·cm^2^ values reached by cells on day 10 under no ALI and ALI conditions. *** *p* < 0.001 (Pairwise Mann-Whitney tests). Table S1. Results of Color Interference and direct MTT reduction activity of medical devices: Color interference was evaluated by adding 100 µL of each medical device to 900 µL of distilled water. OD values exceeding 0.08 were considered indicative of potential interference with formazan dye absorbance. Direct MTT-reducing activity of the medical devices was assessed by incubating 50 µL of each device with 0.4 mL of 1 mg/mL MTT solution, followed by a visual examination of any color change. Table S2. Comparative classification of proficiency chemicals predicted by SkinEthic™ and RhCE model presented in this study.

## Abbreviations

3D	Three-Dimensional
3Rs	Replacement, Reduction, and Refinement
ALI	Air–Liquid Interface
BSA	Bovine Serum Albumin
CAS	Chemical Abstracts Service
CK-3	Cytokeratin 3
CI	Confidence Interval
CO_2_	Carbon Dioxide
CV%	Coefficient of Variation (percentage)
DAPI	4′,6-Diamidino-2-Phenylindole
DMEM	Dulbecco’s Modified Eagle Medium
DMSO	Dimethyl Sulfoxide
DPBS	Dulbecco’s Phosphate-Buffered Saline
EGF	Epidermal Growth Factor
FBOV	Fondazione Banca Degli Occhi del Veneto
FBS	Fetal Bovine Serum
FDA	Food and Drug Administration
GHS	Globally Harmonized System (of Classification and Labelling of Chemicals)
H&E	Hematoxylin and Eosin
IC_50_	Half Maximal Inhibitory Concentration
IgG	Immunoglobulin G
IM-HCEpiC	Immortalized Human Corneal Epithelial Cells
ISO	International Organization for Standardization
κ (Kappa)	Cohen’s Kappa Coefficient
LLI	Liquid–Liquid Interface
MTT	3-(4,5-Dimethylthiazol-2-yl)-2,5-Diphenyltetrazolium Bromide
OD	Optical Density
OECD	Organisation for Economic Co-operation and Development
OECD TG 492	OECD Test Guideline 492
PBS	Phosphate-Buffered Saline
PET	Polyethylene Terephthalate
QC	Quality Control
RH	Relative Humidity
RhCE	Reconstructed Human Cornea-like Epithelium
R^2^	Coefficient of Determination
RT	Room Temperature
SDS	Sodium Dodecyl Sulfate
SD	Standard Deviation
SV40	Simian Virus 40
TEER	Transepithelial Electrical Resistance
TG	Test Guideline
UN	United Nations
VRM	Validated Reference Method
VRM1	Validated Reference Method 1
VRM2	Validated Reference Method 2
ZO-1	Zona Occludens-1

## References

[1] Lu P., Lai J., Tabata Y., Hsiue G. (2008). A methodology based on the “anterior chamber of rabbit eyes” model for noninvasively determining the biocompatibility of biomaterials in an immune privileged site. J. Biomed. Mater. Res. Part A.

[2] Pellevoisin C., Coleman K.P., Hoffmann S. (2022). ISO 10993-23 In vitro irritation testing for medical devices: Substantiating applicability to mild irritants and non-extractables. Toxicol. In Vitro.

[3] Ludwig P.E., Lopez M.J., Sevensma K.E. (2025). Anatomy, Head and Neck, Eye Cornea. StatPearls.

[4] Sridhar M.S. (2018). Anatomy of cornea and ocular surface. Indian J. Ophthalmol..

[5] Eghrari A.O., Riazuddin S.A., Gottsch J.D. (2015). Overview of the Cornea. Progress in Molecular Biology and Translational Science.

[6] Kaluzhny Y., Klausner M. (2021). In vitro reconstructed 3D corneal tissue models for ocular toxicology and ophthalmic drug development. In Vitro Cell. Dev. Biol.-Anim..

[7] OECD (2025). Reconstructed Human Cornea-Like Epithelium (RhCE) Test Method for Identifying Chemicals Not Requiring Classification and Labelling for Eye Irritation or Serious Eye Damage.

[8] OECD (2023). Acute Eye Irritation/Corrosion.

[9] Wilhelmus K.R. (2001). The Draize Eye Test. Surv. Ophthalmol..

[10] Scott L., Eskes C., Hoffmann S., Adriaens E., Alepée N., Bufo M., Clothier R., Facchini D., Faller C., Guest R. (2010). A proposed eye irritation testing strategy to reduce and replace in vivo studies using Bottom-Up and Top-Down approaches. Toxicol. In Vitro.

[11] Russell W.M.S., Burch R.L. (1959). The principles of humane experimental technique. MJA.

[12] OECD (2023). Isolated Chicken Eye Test Method for Identifying i) Chemicals Inducing Serious Eye Damage and ii) Chemicals Not Requiring Classification for Eye Irritation or Serious Eye Damage.

[13] OECD (2025). Bovine Corneal Opacity and Permeability Test Method for Identifying i) Chemicals Inducing Serious Eye Damage and ii) Chemicals Not Requiring Classification for Eye Irritation or Serious Eye Damage.

[14] Chang J.E., Basu S.K., Lee V.H. (2000). Air-interface condition promotes the formation of tight corneal epithelial cell layers for drug transport studies. Pharm. Res..

[15] Rönkkö S., Vellonen K.-S., Järvinen K., Toropainen E., Urtti A. (2016). Human corneal cell culture models for drug toxicity studies. Drug Deliv. Transl. Res..

[16] United Nations (2023). Globally Harmonized System of Classification and Labelling of Chemicals (GHS).

[17] OECD (2025). Short Time Exposure In Vitro Test Method for Identifying i) Chemicals Inducing Serious Eye Damage and ii) Chemicals Not Requiring Classification for Eye Irritation or Serious Eye Damage.

[18] OECD (2023). Fluorescein Leakage Test Method for Identifying Ocular Corrosives and Severe Irritants.

[19] Åhs E., Barroso J., Batista Leite S., Berggren E., Campia I., Carpi D., Casati S., Coecke S., Corvi R., Deceuninck P. (2022). Non-Animal Methods in Science and Regulation: EURL ECVAM Status Report 2021.

[20] Zuang V., Dura A., Ahs L.E., Barroso J., Batista L.S., Berggren E., Bopp S., Campia I., Carpi D., Casati S. (2022). Non-Animal Methods in Science and Regulation.

[21] Greco D., Vellonen K.-S., Turner H.C., Häkli M., Tervo T., Auvinen P., Wolosin J.M., Urtti A. (2010). Gene expression analysis in SV-40 immortalized human corneal epithelial cells cultured with an air-liquid interface. Mol. Vis..

[22] Robertson D.M., Kalangara J.P., Baucom R.B., Petroll W.M., Cavanagh H.D. (2011). A Reconstituted Telomerase-Immortalized Human Corneal Epithelium In Vivo: A Pilot Study. Curr. Eye Res..

[23] OECD (2024). Reconstructed Human Cornea-Like Epithelium (RHCE) Test Method for Eye Hazard Identification.

[24] Iversen P.W., Beck B., Chen Y.-F., Dere W., Devanarayan V., Eastwood B.J., Farmen M.W., Iturria S.J., Montrose C., Moore R.A. (2004). HTS Assay Validation. Assay Guidance Manual [Internet].

[25] Toropainen E., Ranta V.-P., Talvitie A., Suhonen P., Urtti A. (2001). Culture Model of Human Corneal Epithelium for Prediction of Ocular Drug Absorption. Investig. Ophthalmol. Vis. Sci..

[26] Kinoshita S., Adachi W., Sotozono C., Nishida K., Yokoi N., Quantock A.J., Okubo K. (2001). Characteristics of the Human Ocular Surface Epithelium. Prog. Retin. Eye Res..

[27] Wang Y., Chen M., Wolosin J.M. (1993). ZO-1 In Corneal Epithelium; Stratal Distribution and Synthesis Induction by Outer Cell Removal. Exp. Eye Res..

[28] Hahne M., Reichl S. (2011). Development of a serum-free human cornea construct for in vitro drug absorption studies: The influence of varying cultivation parameters on barrier characteristics. Int. J. Pharm..

[29] Kaluzhny Y., Kinuthia M.W., Truong T., Lapointe A.M., Hayden P., Klausner M. (2018). New Human Organotypic Corneal Tissue Model for Ophthalmic Drug Delivery Studies. Investig. Opthalmol. Vis. Sci..

[30] Reichl S. (2004). Human corneal equivalent as cell culture model for in vitro drug permeation studies. Br. J. Ophthalmol..

[31] Srinivasan B., Kolli A.R., Esch M.B., Abaci H.E., Shuler M.L., Hickman J.J. (2015). TEER Measurement Techniques for In Vitro Barrier Model Systems. SLAS Technol..

[32] 1-Ethyl-3-Methylimidazolium Ethylsulfate|C8H16N2O4S|CID 12095229—PubChem. https://pubchem.ncbi.nlm.nih.gov/compound/1-Ethyl-3-methylimidazolium-ethylsulfate.

[33] (2025). Biological Evaluation of Medical Devices Part 1: Requirements and General Principles for the Evaluation of Biological Safety Within a Risk Management Process.

