# Supplementary material for: Development and Validation of an In Vitro Ocular Irritation Test for Ophthalmic Medical Devices with a Novel Reconstructed Human Corneal Epithelium Model
Source: Bioengineering (Basel). 2026 May 11;13(5):545. doi: 10.3390/bioengineering13050545 (PMC13203266; doi:10.3390/bioengineering13050545)
Supplement: Supplementary file 1 [file bioengineering-13-00545-s001.zip › bioengineering-4268824-supplementary.pdf]

Supplementary Materials

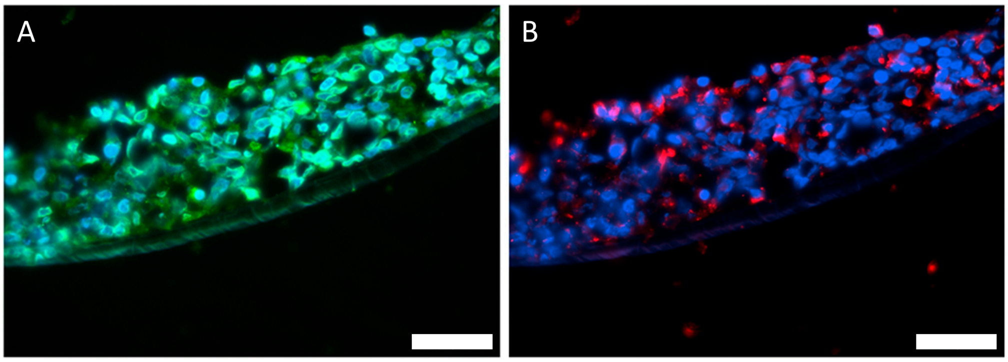

Supplementary Figure S1. Cross-sectional images of RhCE tissue model cultured for a total of 8 days. Immunohistochemical staining for (A) Claudin-3 (green) and (B) Occludin (red) in 5 µm sections. Nuclei were stained with DAPI (blue) Scale bar: 50 µm.

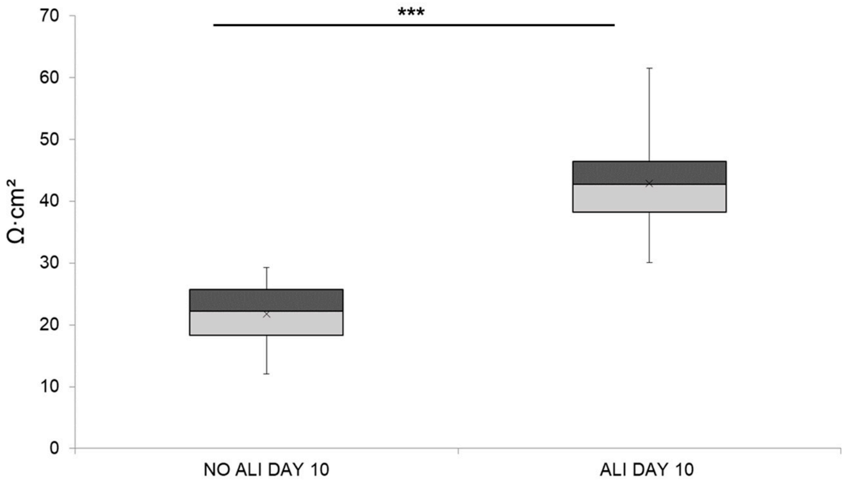

Supplementary Figure S2. Comparison of average TEER (average ± standard deviation, expressed in Ω·cm² values reached by cells on day 10 under no ALI and ALI conditions. *** $p < 0.001$ (Pairwise Mann-Whitney tests).

Supplementary Table S1. Results of Color Interference and direct MTT reduction activity of medical devices: Color interference was evaluated by adding 100 μL of each medical device to 900 μL of distilled water. OD values exceeding 0.08 were considered indicative of potential interference with formazan dye absorbance. Direct MTT-reducing activity of the medical devices was assessed by incubating 50 μL of each device with 0.4 mL of 1 mg/mL MTT solution, followed by a visual examination of any color change.

	**Color interference (OD > 0.08)**				**Direct MTT reduction**	
**Medical Device**	**OD^{550nm}**	**OD^{570nm}**	**OD^{590nm}**	**Color Interference? (Yes/No)**	**Purple coloration observed? (Yes/No)**	**MTT reducer?**
OCIGEL	0.01	0.00	0.00	No	No	No
eyeDRO	0.00	0.00	0.00	No	No	No
PSS-L	0.00	0.00	0.00	No	No	No
TISSUE-C	0.02	0.02	0.00	No	No	No
CARRY-C	0.02	0.02	0.00	No	No	No
Eusol-C	0.03	0.02	0.00	No	No	No
KERASAVE	0.00	0.00	0.00	No	No	No
XTRA4	0.02	0.01	0.00	No	No	No

Supplementary Table S2. Comparative classification of proficiency chemicals predicted by SkinEthic™ and RhCE model presented in this study.

Chemical Name	CAS	Physical state	RhCE model Prediction	SkinEthic™ Prediction
Methylthioglycolate	2365-48-2	L	No prediction can be made	No prediction can be made
Hydroxyethyl acrylate	818-61-1	L	No prediction can be made	No prediction can be made
2,5-Dimethyl-2,5-hexanediol	110-03-2	S	No prediction can be made	No prediction can be made
Sodium oxalate	62-76-0	S	No prediction can be made	No prediction can be made
2,4,11,13Tetraazatetradecanediimidamide, N,N''bis(4-chlorophenyl)-3,12diimino-, di-D- gluconate (20%, aqueous)	18472-51-0	L	No prediction can be made	No prediction can be made
Sodium benzoate	532-32-1	S	No prediction can be made	No prediction can be made
Diethyl toluamide	134-62-3	L	No prediction can be made	No prediction can be made
2,2-Dimethyl-3methylenebicyclo [2.2.1] heptane (CAMPHENE)	79-92-5	S	No prediction can be made	No prediction can be made
1-Ethyl-3methylimidazolium ethylsulphate	342573-75-5	L	No prediction can be made	Non-irritant
Dicaprylyl ether	629-82-3	L	Non-irritant	Non-irritant
Piperonyl butoxide	51-03-6	L	Non-irritant	Non-irritant
Castor oil	61788-85-0	Viscous	Non-irritant	Non-irritant
1-(4-Chlorophenyl)-3- (3,4dichlorophenyl) urea	101-20-2	S	Non-irritant	Non-irritant
2,2'-Methylene-bis-(6-(2Hbenzotriazol-2- yl)-4(1,1,3,3tetramethylbutyl)-phenol)	103597-451	S	Non-irritant	Non-irritant
Potassium tetrafluoroborate	14075-53-7	S	Non-irritant	Non-irritant
